# Cognitive dysfunction during mild to moderate migraine attacks: potential implications for presenteeism

**DOI:** 10.1186/s12883-026-04782-z

**Published:** 2026-03-04

**Authors:** Yoji Yamaguchi, Masaaki Kohta, Kenji Ishikawa, Yasuo Naito, Michio Yamaguchi, Yusuke Ikeuchi, Hiroyasu Shose, Kazuhiro Tanaka, Atsushi Fujita, Takashi Sasayama

**Affiliations:** 1 Department of Neurosurgery, Kobe University Graduate School of Medicine, 7-5-1 Kusunoki-cho, Chuo-ku, Kobe, Hyogo 650-0017 Japan; 2 Yamaguchi Clinic, Nishinomiya, Hyogo Japan; 3 Department of Occupational Therapy, Osaka University of Human Sciences, Settsu, Osaka Japan; 4 Department of Occupational Therapy, Osaka Metropolitan University Graduate School of Rehabilitation Science, Osaka, Osaka Japan

**Keywords:** Migraine, Cognitive function, Presenteeism, Digit Cancellation Test (D-CAT), Trail Making Test (TMT)

## Abstract

**Background:**

Migraine-related presenteeism is a global concern, arising when individuals attend work during a headache attack, resulting in reduced performance. To clarify this issue, we investigated the effect of the presence and severity of headache attacks on cognitive function in patients with migraine.

**Methods:**

We conducted a retrospective analysis of prospectively collected cohort data from April to December 2019, enrolling participants aged 18–80 years who met the ICHD-3 criteria for migraine. Participants were categorized into four groups based on headache (HA) intensity at the time of their clinic visit: no HA, mild, moderate, or severe. Cognitive function was assessed using the Digit Cancellation Test (D-CAT) and the Trail Making Test (TMT). Patients with severe HAs were excluded, as pain relief treatment was prioritized. Statistical comparisons were conducted across the remaining groups.

**Results:**

A total of 259 patients were analyzed, including 125 without HAs (HA (−)) and 134 with HAs (HA (+)), of whom 79 were mild and 55 were moderate. The HA (+) group showed significantly lower D-CAT scores compared with the HA (−) group (*p* < 0.05), while TMT scores did not differ significantly between the two groups. Within the HA (+) group, no significant differences in D-CAT or TMT scores were observed between patients with mild and moderate HAs. Similarly, cognitive scores did not differ between migraine patients with and without aura.

**Conclusion:**

Migraine patients experiencing a headache attack had reduced cognitive function compared with HA-free migraine patients, and this dysfunction was independent of HA severity. These findings suggest that the presence of a headache attack itself – regardless of its severity – may impair cognitive function and have potential implications for work efficiency and presenteeism.

**Supplementary Information:**

The online version contains supplementary material available at 10.1186/s12883-026-04782-z.

## Background

Migraine is a neurological disorder characterized by recurrent attacks that significantly reduce quality of life. Globally, migraine affects an estimated 11.6% of the population, with over 800 million individuals suffering from the condition [1]. Migraine is currently ranked as the third leading cause of years lived with disability worldwide [2]. In addition to its physical burden, migraine is associated with cognitive impairment and results in substantial socio-economic consequences, including reduced productivity, efficiency, and work performance. In Japan, the prevalence of migraine is estimated to be 6%–8.4% [3, 4], with approximately 8 million people affected [5]. This results in economic losses of up to 3 billion USD (approximately 360 billion yen) annually due to reduced productivity [5].

The economic impact of migraine is thought to be greater for presenteeism—when individuals are at work but unable to perform effectively—than for absenteeism, when individuals are unable to attend work. Studies indicate that 28%–39% of migraine patients report reduced productivity at work or school because of their headaches (HAs) [6, 7]. Moreover, 80% of migraine patients experience associated symptoms, such as fatigue, irritability, difficulty concentrating, stiff neck, phonophobia, and nausea [8]. These symptoms are believed to further reduce productivity.

Understanding the mechanisms behind reduced productivity in migraine patients requires careful examination of how migraine affects cognitive function. Numerous studies have investigated the relationship between migraine and cognitive function, including research conducted during migraine attacks [9–12], during interictal periods [13–15], and in relation to long-term disease duration [16]. Some reports focusing on the ictal period have shown that cognitive function is impaired during migraine attacks [9–12] and subsequently improves with appropriate pharmacological treatment [9, 11]. However, these studies have not evaluated cognitive function by classifying patients according to the severity of their headache attacks. When discussing presenteeism, we considered that patients with severe headache attacks, which correspond to absenteeism, should be excluded. Instead, we focused on those experiencing headache attacks that are mild or moderate in severity, as they are still capable of attending work and performing their duties. In this study, we assessed whether patients were experiencing a migraine attack at the time of their clinic visit. If an attack was present, patients were further classified according to headache severity. Cognitive function was then evaluated in those with mild to moderate headaches, as these cases were regarded as representative of presenteeism.

## Methods

We performed a retrospective analysis of prospectively collected cohort data, with participants enrolled between April and December 2019. The inclusion criteria were individuals aged 18–80 years who had been diagnosed with migraine with aura (MA) or without aura (MO). To confirm the diagnosis, all participants underwent a clinical assessment conducted by two Japanese Headache Society board-certified HA specialists (Y.Y. and M.Y.). The diagnosis followed the guidelines of the International Classification of Headache Disorders, third edition (ICHD-3). No separate structured interview instrument was used. Additionally, head computed tomography scans were performed to rule out organic intracranial diseases. Participants with clinically apparent cognitive impairment, including dementia or other neurocognitive disorders, were excluded based on routine clinical evaluation and review of medical records.

At the time of their clinic visit, the severity of each participant’s HA was assessed and classified into four categories:

- No HA: Pain-free
- Mild HA: Noticeable pain that does not interfere with daily activities
- Moderate HA: Pain that interferes with daily activities but still allows the patient to function
- Severe HA: Intense pain that makes daily activities extremely difficult

Participants with severe HAs were excluded from cognitive assessment, as immediate pain relief was prioritized. All cognitive assessments were conducted during the headache attack phase and not during the prodromal or aura phases. In addition, participants with mild to moderate HAs were evaluated before the administration of any acute headache medication, and thus were assessed without the influence of acute pharmacological treatment. Cognitive function was evaluated only in participants with no, mild, or moderate HAs using standardized neuropsychological tests as part of the study. As this was a retrospective analysis of a prospectively collected cohort, no formal a priori sample size calculation was performed. All eligible participants enrolled during the study period were included.

### Evaluation of cognitive function

In this study, we evaluated cognitive function using both the Digit Cancellation Test (D-CAT) and the Trail Making Test (TMT).

### Digit Cancellation Test (D-CAT)

The D-CAT was used to assess attentional function [17]. In this test, participants were given a sheet with 12 rows of 50 digits each, totaling 600 digits per sheet. Each row contained five sets of digits (0 to 9) arranged in random order. The task requires participants to locate and mark a specific target digit(s) by placing slashes through them as quickly and accurately as possible within 1 min. The test consists of three separate trials: In trial one (D-CAT1), participants search for a single target digit; in trial two (D-CAT2), participants search for two target digits; in trial three (D-CAT3), participants search for three target digits. Each trial uses a different arrangement of digits, with a 1-minute break between trials. The main measure of performance is the total number of digits scanned, reflecting key cognitive abilities such as information processing speed, focused attention, and sustained attention [17].

### Trail Making Test (TMT)

The TMT is a well-established tool for assessing cognitive function and consists of two types, TMT-A and TMT-B. TMT-A measures visual scanning, graphomotor speed, and visuomotor processing speed by requiring participants to connect numbers in ascending order. TMT-B assesses these abilities along with executive function by prompting participants to alternate between numbers and letters in sequence [18]. Together, the two parts provide a comprehensive evaluation of cognitive performance.

### Statistical analysis

To evaluate differences in cognitive function based on the presence or absence of headache attacks, participants were divided into two groups: the HA (−) group (no HA) and the HA (+) group (mild or moderate HA). Further comparisons were conducted within the HA (+) group by comparing the mild and moderate subgroups. Analyses were also performed to compare patients with MO and MA in the HA (+) group. Data distribution was assessed, and because several continuous variables deviated from normality, continuous data were presented as median (interquartile range [IQR]). The Mann–Whitney U test was used to identify statistically significant differences between groups. Fisher’s exact test was also employed to compare the proportions of categorical variables between the two groups. Statistical significance was set at *p* < 0.05 for all analyses. For nonparametric group comparisons, effect sizes and 95% confidence intervals were estimated using the Hodges–Lehmann method. In addition, multiple linear regression analyses were performed for the primary cognitive outcomes to adjust for age, with headache status and age included as independent variables. As a sensitivity analysis, analyses were restricted to participants aged ≤ 60 years to better reflect the working-age population relevant to presenteeism. Subgroup analyses (mild vs. moderate HA and MO vs. MA) were considered exploratory, and no formal adjustment for multiple comparisons was applied.

## Results

### Patients

A total of 259 patients were included in the study. Of these, 125 were classified as having no HA, 79 as having mild HA, and 55 as having moderate HA. Thus, 125 patients were categorized into the HA (−) group (no HA), and 134 into the HA (+) group (mild or moderate HA). The median age was 37 years (IQR: 28–47) in the HA (−) group and 38 years (IQR: 29–45) in the HA (+) group, with no statistically significant difference (*p* = 0.67). The HA (−) group included 22 males (18%), and the HA (+) group included 24 males (18%) (*p* = 0.99). The median disease duration was 16 years (IQR: 8–27) in the HA (−) group and 18 years (IQR: 8–27) in the HA (+) group, which also represented no significant difference (*p* = 0.95). The HA (−) group comprised 103 patients with MO and 22 with MA, while the HA (+) group comprised 112 patients with MO and 22 with MA (*p* = 0.87). Associated symptoms, including nausea, photophobia, phonophobia, osmophobia, vertigo, dizziness, and tinnitus, showed no statistically significant differences between the two groups (Table 1).

**Table 1:** Clinical characteristics of HA (−) and HA (+) groups

	HA (−)	HA (+)	*p* value
*N*	125	134	
Age (y.o.)	37 (28–47)	38 (29–45)	0.67
Male (%)	22 (18)	24 (18)	0.99
Disease duration (y)	16 (8–27)	18 (8–27)	0.95
MO/MA	103/22	112/22	0.87
Nausea (%)	74 (59)	83 (62)	0.70
Photophobia (%)	56 (45)	57 (43)	0.80
Phonophobia (%)	62 (50)	65 (49)	0.90
Osmophobia (%)	18 (14)	19 (14)	0.99
Vertigo (%)	19 (15)	21 (16)	0.99
Dizziness (%)	39 (31)	42 (31)	0.99
Tinnitus (%)	10 (8)	13 (10)	0.67

Mann–Whitney *U* test, Fisher’s exact test. Values are reported as median (IQR) or number (%). HA (−), without headache; HA (+), with headache; MA, migraine with aura; MO, migraine without aura. Associated symptoms were determined based on patients’ headache history and clinical interviews and do not necessarily reflect symptoms present at the time of cognitive assessment

### Cognitive function of HA (−) and HA (+) groups

**Fig. 1:**
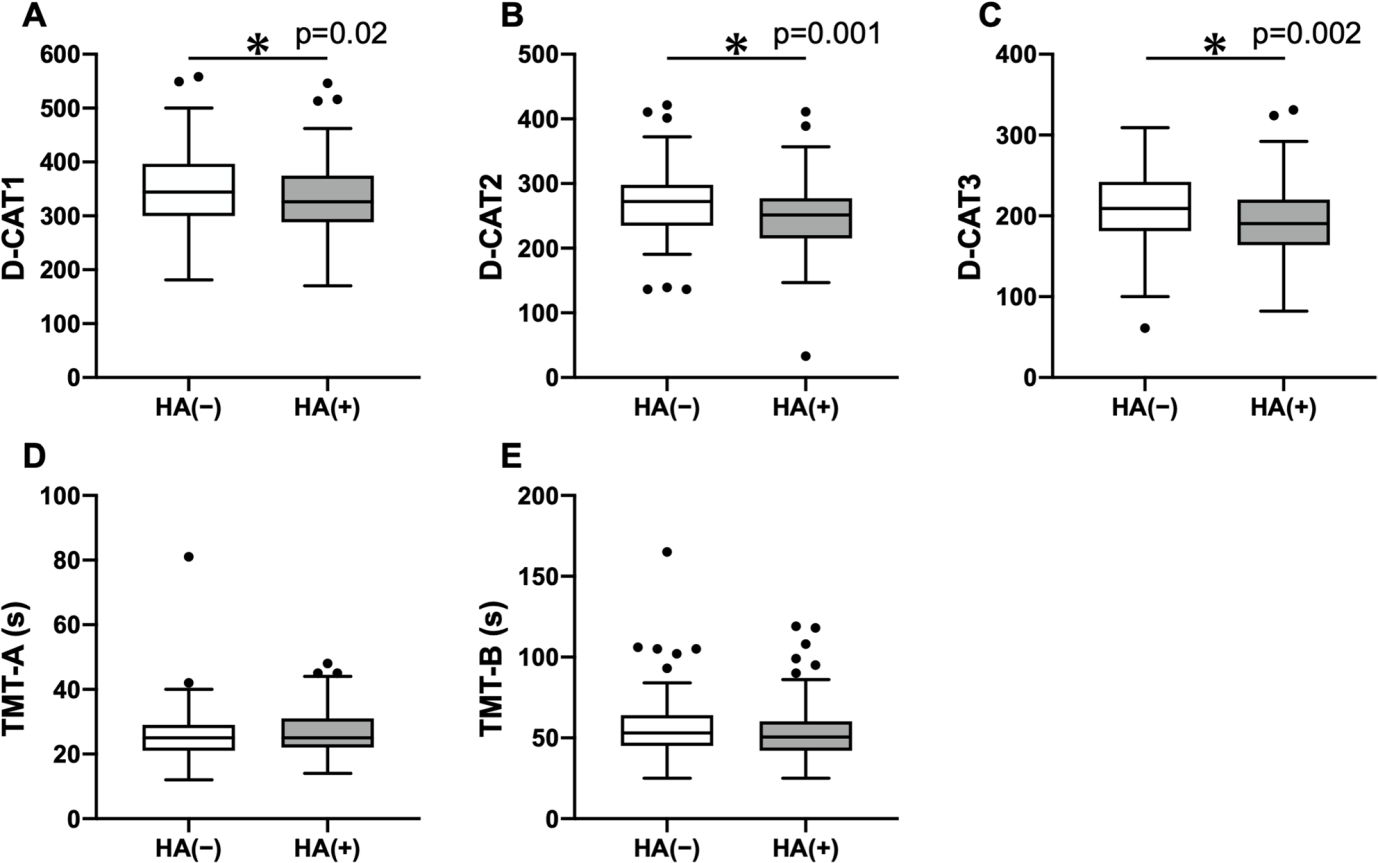
Box-and-whisker plots showing that D-CAT1 (Fig. **A**), D-CAT2 (Fig. **B**), and D-CAT3 (Fig. **C**) scores were significantly lower in the HA (+) group compared with the HA (−) group. No significant differences were observed between the two groups for either TMT-A (Fig. **D**) or TMT-B (Fig. **E**). Boxes represent the interquartile range, center lines indicate medians, and whiskers extend to 1.5× the interquartile range. * indicates *p* < 0.05. Sample sizes were HA (−), *n* = 125; HA (+), *n* = 134. D-CAT, Digit Cancellation Test; HA (−), without headache; HA (+), with headache; TMT, Trail Making Test

D-CAT scores were compared between the HA (−) and HA (+) groups. Across all trials, the HA (+) group demonstrated significantly lower D-CAT scores compared with the HA (−) group. The median D-CAT1 scores were 326 (IQR: 288–375) in the HA (+) group and 344 (IQR: 300–397) in the HA (−) group (*p* = 0.02), corresponding to a median difference of − 21 (95% CI, − 38 to − 2); the median D-CAT2 scores were 252 (IQR: 215–277) and 272 (IQR: 235–298), respectively (*p* = 0.001), with a median difference of − 19 (95% CI, − 30 to − 8); and the median D-CAT3 scores were 191 (IQR: 164–220) and 209 (IQR: 181–242), respectively (*p* = 0.002), with a median difference of − 16 (95% CI, − 26 to − 6) (Fig. 1, Supplemental Table 1). To account for potential confounding by age, additional multiple linear regression analyses adjusting for age were performed. Headache status remained a significant independent predictor of lower performance on D-CAT1 (β = −20.2, 95% CI, − 37.1 to − 3.3; *p* = 0.02), D-CAT2 (β = −19.8, 95% CI, − 32.0 to − 7.7; *p* = 0.002), and D-CAT3 (β = −15.2, 95% CI, − 25.5 to − 4.9; *p* = 0.004), whereas age itself was not significantly associated with any D-CAT measures. For the TMT, the median TMT-A times were 25 (IQR: 22–31) s in the HA (+) group and 25 (IQR: 21–29) s in the HA (−) group (*p* = 0.26), corresponding to a median difference of − 1 s (95% CI, − 3 to 1), while the median TMT-B times were 51 (IQR: 42–60) s in the HA (+) and 53 (IQR: 45–64) s in the HA (−) (*p* = 0.27), corresponding to a median difference of 2 s (95% CI, − 2 to 6). No significant differences were observed between the two groups for either TMT measure (Fig. 1, Supplemental Table 1). To address concerns regarding age and occupational relevance, we performed a sensitivity analysis restricted to participants aged ≤ 60 years. In this subgroup, the HA (+) group continued to show significantly lower D-CAT performance than the HA (−) group. Median D-CAT1 scores were 326 (IQR: 288–375) in the HA (+) group and 350 (IQR: 300–403) in the HA (−**)** group (*p* = 0.02), with a median difference of − 22 (95% CI, − 40 to − 4). Median D-CAT2 scores were 252 (IQR: 215–277) and 274 (IQR: 235–300), respectively (*p* = 0.001), with a median difference of − 21 (95% CI, − 31 to − 9), and median D-CAT3 scores were 191 (IQR: 164–220) and 209 (IQR: 182–243), respectively (*p* = 0.001), with a median difference of − 18 (95% CI, − 28 to − 7) (Supplemental Table 2). No significant differences were observed for TMT-A or TMT-B in the ≤ 60-year subgroup (both *p* > 0.1).

### Cognitive function of mild HA and moderate HA groups

In the comparison between the mild (*n* = 79) and moderate (*n* = 55) HA groups, the median age was 37 (IQR: 26–46) years and 38 (IQR: 31–45) years, respectively (*p* = 0.89). The number of males was 17 (22%) and 7 (13%) in the mild and moderate HA groups, respectively (*p* = 0.25). The median disease duration was 16 (IQR: 7–27) years and 19 (IQR: 9–28) years for the mild and moderate HA groups, respectively (*p* = 0.83). No significant differences were observed for any of these variables. The MO/MA ratio was 65/14 for the mild HA group and 47/8 for the moderate HA group, with no significant difference (*p* = 0.81). Associated symptoms, including nausea, photophobia, phonophobia, osmophobia, vertigo, dizziness, and tinnitus, showed no statistically significant differences between the mild and moderate HA groups (Table 2).

**Table 2:** Clinical characteristics of mild HA and moderate HA groups

	Mild HA	Moderate HA	*p* value
*N*	79	55	
Age (y.o.)	37 (26–46)	38 (31–45)	0.89
Male (%)	17 (22)	7 (13)	0.25
Disease duration (y)	16 (7–27)	19 (9–28)	0.83
MO/MA	65/14	47/8	0.81
Nausea (%)	50 (63)	33 (60)	0.72
Photophobia (%)	37 (47)	20 (36)	0.29
Phonophobia (%)	38 (48)	27 (49)	0.99
Osmophobia (%)	10 (13)	9 (16)	0.62
Vertigo (%)	12 (15)	9 (16)	0.99
Dizziness (%)	26 (33)	16 (29)	0.71
Tinnitus (%)	10 (13)	3 (5)	0.24

Mann–Whitney *U* test, Fisher’s exact test. Values are reported as median (IQR) or number (%). HA, headache; MA, migraine with aura; MO, migraine without aura. Associated symptoms were determined based on patients’ headache history and clinical interviews and do not necessarily reflect symptoms present at the time of cognitive assessment

**Fig. 2:**
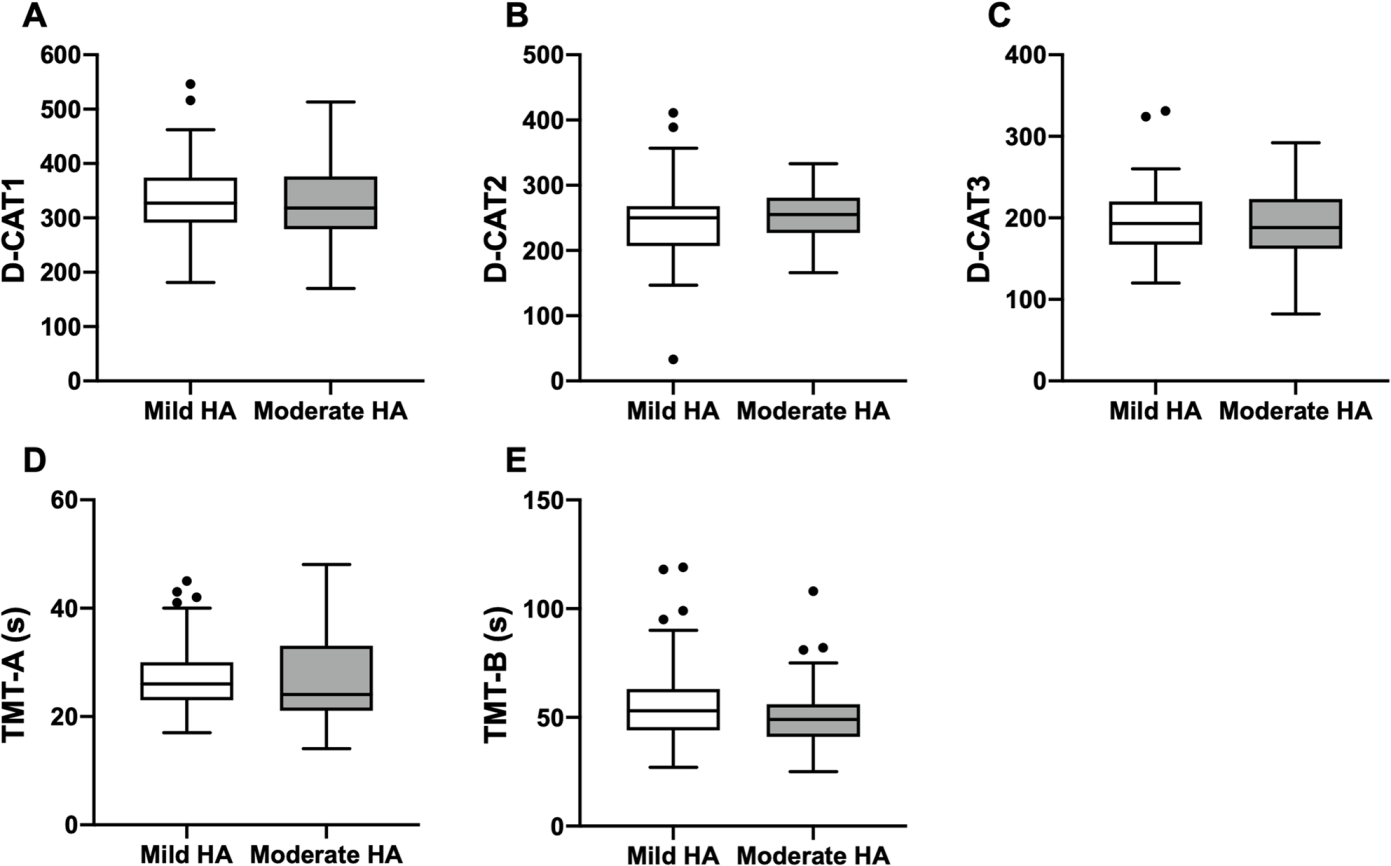
Box-and-whisker plots showing no significant differences between the mild HA group and the moderate HA group for D-CAT1 (Fig. **A**), D-CAT2 (Fig. **B**), and D-CAT3 (Fig. **C**). Similarly, no significant differences were observed for TMT-A (Fig. **D**) and TMT-B (Fig. **E**). Boxes represent the interquartile range, center lines indicate medians, and whiskers extend to 1.5× the interquartile range. Sample sizes were mild HA, *n* = 79; moderate HA, *n* = 55. D-CAT, Digit Cancellation Test; HA, headache; TMT, Trail Making Test

D-CAT scores were compared between the mild and moderate HA groups. The median D-CAT1 scores were 327 (IQR: 291–374) and 318 (IQR: 279–376), respectively (*p* = 0.58); the median D-CAT2 scores were 250 (IQR: 207–268) and 255 (IQR: 227–281) (*p* = 0.29); and the median D-CAT3 scores were 193 (IQR: 167–220) and 188 (IQR: 162–223) (*p* = 0.81). None of these differences were statistically significant. For the TMT, the median TMT-A times were 26 (IQR: 23–30) s in the mild HA group and 24 (IQR: 21–33) s in the moderate HA group (*p* = 0.54). The median TMT-B times were 53 (IQR: 44–63) s in the mild HA group and 49 (IQR: 41–56) s in the moderate HA group (*p* = 0.21). No significant differences were observed between the two groups for either TMT measure (Fig. 2, Supplemental Table 3).

### Cognitive function of MO and MA in the HA (+) group

A total of 112 patients with MO and 22 patients with MA in the HA (+) group were evaluated. The median age was 38 (IQR: 29–45) years in the MO group and 36 (IQR: 29–49) years in the MA group (*p* = 0.91). The number of males was 21 (19%) in the MO group and 3 (14%) in the MA group (*p* = 0.76). The median disease duration was 19 (IQR: 8–27) years for the MO group and 17 (IQR: 6–30) years for the MA group (*p* = 0.93). Among associated symptoms, osmophobia was significantly more frequent in the MA group than in the MO group, whereas no significant differences were observed for nausea, photophobia, phonophobia, vertigo, dizziness, or tinnitus (Table 3).

**Table 3:** Clinical characteristics of MO and MA patients in the HA (+) group

	MO	MA	*p* value
*N*	112	22	
Age (y.o.)	38 (29–45)	36 (29–49)	0.91
Male (%)	21 (19)	3 (14)	0.76
Disease duration (y)	19 (8–27)	17 (6–30)	0.93
Nausea (%)	67 (60)	16 (73)	0.34
Photophobia (%)	45 (40)	12 (55)	0.24
Phonophobia (%)	53 (47)	12 (55)	0.64
Osmophobia (%)	12 (11)	7 (32)	0.02
Vertigo (%)	15 (13)	6 (27)	0.11
Dizziness (%)	34 (30)	8 (36)	0.62
Tinnitus (%)	9 (8)	4 (18)	0.23

Mann–Whitney *U* test, Fisher’s exact test. Values are reported as median (IQR) or number (%). HA, headache; MA, migraine with aura; MO, migraine without aura. Associated symptoms were determined based on patients’ headache history and clinical interviews and do not necessarily reflect symptoms present at the time of cognitive assessment

**Fig. 3:**
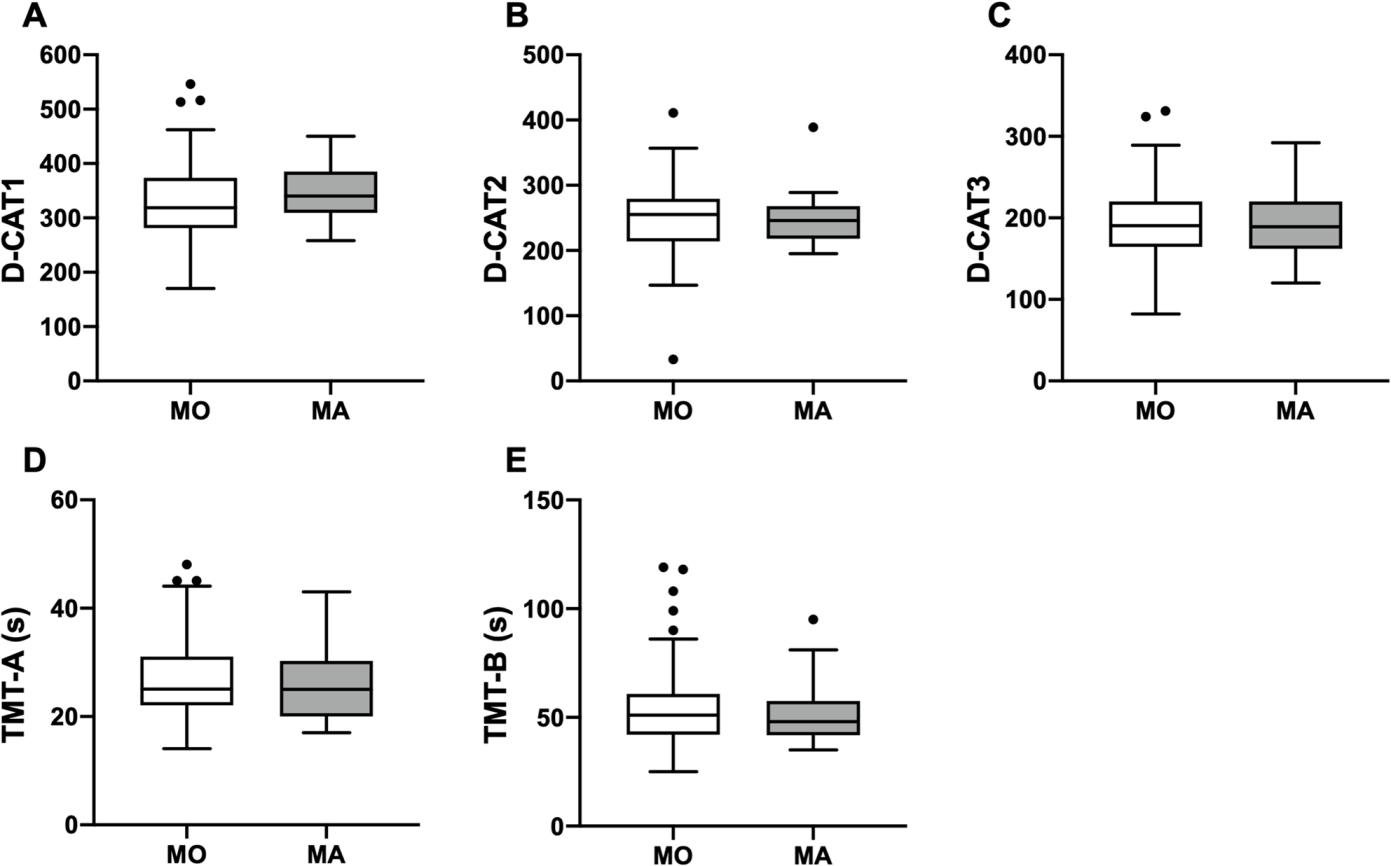
Box-and-whisker plots showing no significant differences between MO and MA patients in the HA (+) group for D-CAT1 (Fig. **A**), D-CAT2 (Fig. **B**), and D-CAT3 (Fig. **C**). Similarly, no significant differences were observed for TMT-A (Fig. **D**) and TMT-B (Fig. **E**). Boxes represent the interquartile range, center lines indicate medians, and whiskers extend to 1.5× the interquartile range. Sample sizes were MO, *n* = 112; MA, *n* = 22. D-CAT, Digit Cancellation Test; HA, headache; MA, migraine with aura; MO, migraine without aura; TMT, Trail Making Test

D-CAT scores were compared between the MO and MA groups. The median D-CAT1 scores were 319 (IQR: 281–374) for the MO group and 340 (IQR: 309–385) for the MA group (*p* = 0.16); the median D-CAT2 scores were 255 (IQR: 214–280) and 246 (IQR: 218–268), respectively (*p* = 0.62); and the median D-CAT3 scores were 191 (IQR: 165–220) and 189 (IQR: 162–220), respectively (*p* = 0.79). None of these differences were statistically significant. For the TMT, the median TMT-A times were 25 (IQR: 22–31) s in the MO group and 25 (IQR: 20–30) s in the MA group (*p* = 0.56), while the median TMT-B times were 51 (IQR: 42–61) s and 48 (IQR: 42–58) s, respectively (*p* = 0.89). No significant differences were observed between the two groups for either TMT measure (Fig. 3, Supplemental Table 4).

## Discussion

This study revealed that migraine patients experiencing a mild to moderate headache attack had reduced cognitive function compared with HA-free migraine patients. Moreover, the degree of cognitive dysfunction was not significantly affected by whether the HA was mild or moderate. These findings suggest that working while experiencing even a mild to moderate migraine attack may impair cognitive performance and reduce work efficiency, with potential implications for migraine-related presenteeism.

Migraine is associated with substantial economic loss, making it a significant public health and social issue. Presenteeism—attending work or school but being unable to perform effectively—has emerged as a significant issue for migraine patients. It has been reported that 89% of migraine-related productivity loss is due to presenteeism [19]. Migraine is known to impair work performance, with previous reports indicating that 28% to 39% of migraine patients experience reduced productivity [6, 7, 20]. Furthermore, approximately one-third of migraine attacks occur on workdays, and two-thirds of these result in substantial productivity losses [21]. It has also been suggested that productivity loss due to presenteeism exceeds that caused by absenteeism [22, 23].

Our study showed that migraine patients with mild to moderate HAs had lower cognitive function than those without HAs. Previous studies have similarly shown that cognitive function is impaired during migraine attacks [9–12], and this dysfunction has been associated with reduced work efficiency and presenteeism. However, prior research has not typically classified patients by HA severity, and likely included individuals with severe symptoms. In our study, we categorized patients according to HA severity. Patients with severe symptoms were excluded from the analysis, as we considered them to represent absenteeism and prioritized their treatment due to the intensity of their pain. Our evaluation therefore focused on patients with mild to moderate HAs. To our knowledge, this is the first study to assess cognitive function specifically during mild to moderate HAs, which may have implications for presenteeism. To further support the relevance of these findings to working-age individuals, we performed a sensitivity analysis restricted to participants aged ≤ 60 years. The main results remained consistent in this subgroup, with migraine patients experiencing mild to moderate HAs continuing to show significantly lower cognitive performance compared with HA-free patients. These findings suggest that the observed cognitive dysfunction during mild to moderate migraine attacks is not driven by older participants and is also applicable to a population more likely to be actively employed.

Our study also found no significant difference in cognitive function between patients with mild and moderate HAs, suggesting that HA severity may not significantly impact work performance. In contrast, a previous study reported a correlation between migraine severity and reduced work efficiency [24]; however, it relied on self-reported questionnaire data and included patients with severe headaches. In our study, cognitive function was directly assessed using objective, performance-based tests during migraine attacks, which may explain the differing results. Moreover, our evaluations were conducted in an outpatient clinic rather than in an actual workplace setting, which may have influenced the observed outcomes. The absence of a significant difference between mild and moderate HAs may also reflect limited statistical power to detect modest between-group differences.

In our analysis, no significant difference in cognitive function was observed between patients with MO and MA in the HA (+) group. The two subtypes are thought to differ in their underlying pathophysiological mechanisms [25], and previous studies have suggested that MA is associated with a higher risk of developing dementia compared with MO [26]. However, most studies examining the relationship between migraine and cognitive function have not distinguished between MO and MA [25, 27], and to date, no research has directly compared these subtypes during headache attacks. Further large-scale investigations are warranted to clarify potential differences in cognitive function between MO and MA.

We used the D-CAT and TMT to assess cognitive function. Among migraine patients experiencing mild to moderate HAs, D-CAT scores were significantly lower than those of HA-free migraine patients, while TMT scores did not differ significantly between groups. The D-CAT requires participants to select and mark target numbers from a series of listed digits within a fixed time frame. This test evaluates various aspects of prefrontal cortex function, including information processing speed, focused attention, and sustained attention [17]. In contrast, the TMT evaluates different cognitive domains: TMT-A measures visual scanning, graphomotor speed, and visuomotor processing speed, while TMT-B assesses working memory, inhibition control, and executive functions [18]. TMT performance involves not only the inferior medial frontal cortex but also non-frontal brain regions, including the left precentral gyrus, angular gyrus, medial temporal gyrus, and intraparietal sulcus [18]. This broader neural involvement may explain the absence of significant TMT score differences during migraine attacks. Overall, these findings suggest that migraine attacks may primarily impair functions related to the prefrontal cortex.

This study has several limitations. First, it was designed as a retrospective, single-center study, which may limit the generalizability of the findings. To confirm and extend these results, larger prospective studies conducted across multiple centers are warranted. Second, the severity of headache attacks was determined based on patient self-assessment. While this approach reflects the patient’s subjective experience, it lacks objective validation, and future studies should consider incorporating standardized, clinician-administered measures. In addition, headache severity was classified based on patients’ subjective reports without validated numerical pain scales (e.g., VAS or NRS), which may limit the reliability and reproducibility of severity-based subgroup comparisons. Furthermore, patients with severe headache were excluded based on the assumption that they are more likely to experience absenteeism; this may have introduced selection bias. Third, although participants with clinically apparent cognitive impairment were excluded in routine clinical practice, formal cognitive screening was not performed; therefore, subtle pre-existing cognitive deficits cannot be completely ruled out. Ideally, within-subject comparisons between ictal and interictal states would provide a more direct assessment of transient cognitive changes associated with headache attacks; however, the present study was designed to evaluate differences in cognitive performance at the time of clinic visit according to the presence or absence of headache, reflecting real-world clinical practice in which some patients seek medical care only during symptomatic periods. In addition, several important potential confounders, including educational background, psychiatric comorbidities, medication use, sleep status, anxiety, occupation, and migraine chronicity (episodic vs. chronic), were not systematically assessed or adjusted for and may have influenced the observed associations. Presenteeism was not directly measured using validated workplace productivity instruments; thus, the relationship between cognitive performance and presenteeism should be interpreted as inferred rather than directly assessed. Lastly, regarding the D-CAT, we used total performance (the total number of digits processed) as the primary metric, enabling the evaluation of information processing speed, focus, and selective attention [17]. We excluded other D-CAT metrics, such as the omission ratio (the proportion of missed target digits) and the reduction ratio (the rate of performance reduction in D-CAT2 and D-CAT3), which assess sustained attention and tolerance for mental fatigue [17], in order to simplify the analysis in this study. Future research should incorporate these additional measures to allow for a more comprehensive assessment.

## Conclusions

This study demonstrated that migraine patients experiencing a mild to moderate headache attack had reduced cognitive function compared with HA-free migraine patients. Notably, the degree of cognitive dysfunction was not significantly influenced by whether the HA was mild or moderate. These findings suggest that the presence of HA itself, regardless of its severity, may impair cognitive performance, with potential implications for work efficiency and migraine-related presenteeism.

## Supplementary Information

Supplementary Material 1.

Supplementary Material 2.

Supplementary Material 3.

Supplementary Material 4.

## Data Availability

No datasets were generated or analysed during the current study.

## Abbreviations

D-CAT: Digit Cancellation Test
HA: Headache
ICHD-3: International Classification of Headache Disorders, third edition
MA: Migraine with aura
MO: Migraine without aura
TMT: Trail Making Test

## References

[1] Woldeamanuel YW, Cowan RP. Migraine affects 1 in 10 people worldwide featuring recent rise: A systematic review and meta-analysis of community-based studies involving 6 million participants. J Neurol Sci Jan. 2017;15:372:307–15. 10.1016/j.jns.2016.11.071. 10.1016/j.jns.2016.11.071 28017235

[2] Diseases GBD, Injuries C. Global incidence, prevalence, years lived with disability (YLDs), disability-adjusted life-years (DALYs), and healthy life expectancy (HALE) for 371 diseases and injuries in 204 countries and territories and 811 subnational locations, 1990–2021: a systematic analysis for the Global Burden of Disease Study 2021. Lancet May. 2024;18(10440):2133–61. 10.1016/S0140-6736(24)00757-8. 10.1016/S0140-6736(24)00757-8 PMC11122111 38642570

[3] Sakai F, Igarashi H. Prevalence of migraine in Japan: a nationwide survey. Cephalalgia Feb. 1997;17(1):15–22. 10.1046/j.1468-2982.1997.1701015.x. 10.1046/j.1468-2982.1997.1701015.x 9051330

[4] Takeshima T, Wan Q, Zhang Y, et al. Prevalence, burden, and clinical management of migraine in China, Japan, and South Korea: a comprehensive review of the literature. J Headache Pain Dec. 2019;5(1):111. 10.1186/s10194-019-1062-4. 10.1186/s10194-019-1062-4 PMC6896325 31805851

[5] Shimizu T, Sakai F, Miyake H, et al. Disability, quality of life, productivity impairment and employer costs of migraine in the workplace. J Headache Pain Apr. 2021;21(1):29. 10.1186/s10194-021-01243-5. 10.1186/s10194-021-01243-5 PMC8061063 33882816

[6] Lipton RB, Bigal ME, Diamond M, et al. Migraine prevalence, disease burden, and the need for preventive therapy. Neurology. 2007;68(5):343–9. . 10.1212/01.wnl.0000252808.97649.21. 17261680 10.1212/01.wnl.0000252808.97649.21

[7] Wong LP, Alias H, Bhoo-Pathy N, et al. Impact of migraine on workplace productivity and monetary loss: a study of employees in banking sector in Malaysia. J Headache Pain Jun. 2020;8(1):68. 10.1186/s10194-020-01144-z. 10.1186/s10194-020-01144-z PMC7282083 32513174

[8] Charles A. The evolution of a migraine attack - a review of recent evidence. Headache Feb. 2013;53(2):413–9. 10.1111/head.12026. 10.1111/head.12026 23278169

[9] Farmer K, Cady R, Bleiberg J, Reeves D. A pilot study to measure cognitive efficiency during migraine. Headache Sep. 2000;40(8):657–61. 10.1046/j.1526-4610.2000.040008657.x. 10.1046/j.1526-4610.2000.040008657.x 10971662

[10] Meyer JS, Thornby J, Crawford K, Rauch GM. Reversible cognitive decline accompanies migraine and cluster headaches. Headache Sep. 2000;40(8):638–46. 10.1046/j.1526-4610.2000.040008638.x. 10.1046/j.1526-4610.2000.040008638.x 10971660

[11] Farmer K, Cady R, Bleiberg J, et al. Sumatriptan nasal spray and cognitive function during migraine: results of an open-label study. Headache Apr. 2001;41(4):377–84. 10.1046/j.1526-4610.2001.111006377.x. 10.1046/j.1526-4610.2001.111006377.x 11318884

[12] Edwards KR, Rosenthal BL, Farmer KU, Cady RK, Browning R. Evaluation of sumatriptan-naproxen in the treatment of acute migraine: a placebo-controlled, double-blind, cross-over study assessing cognitive function. Headache Apr. 2013;53(4):656–64. 10.1111/head.12052. 10.1111/head.12052 23406052

[13] Martins IP, Gil-Gouveia R, Silva C, Maruta C, Oliveira AG. Migraine, headaches, and cognition. Headache Nov-Dec. 2012;52(10):1471–82. 10.1111/j.1526-4610.2012.02218.x. 10.1111/j.1526-4610.2012.02218.x 22830358

[14] Le Pira F, Reggio E, Quattrocchi G, et al. Executive dysfunctions in migraine with and without aura: what is the role of white matter lesions? Headache Jan. 2014;54(1):125–30. 10.1111/head.12158. 10.1111/head.12158 23808818

[15] Baschi R, Monastero R, Cosentino G, et al. Visuospatial learning is fostered in migraine: evidence by a neuropsychological study. Neurol Sci Nov. 2019;40(11):2343–8. 10.1007/s10072-019-03973-6. 10.1007/s10072-019-03973-6 31250281

[16] Pearson AJ, Chronicle EP, Maylor EA, Bruce LA. Cognitive function is not impaired in people with a long history of migraine: a blinded study. Cephalalgia Jan. 2006;26(1):74–80. 10.1111/j.1468-2982.2005.01001.x. 10.1111/j.1468-2982.2005.01001.x 16396669

[17] Hatta TY, Ito K, Mase Y, Kabasawa M. Reliability and validity of the digit cancellation test, a brief screen of attention. Psychologia. 2012;55:246–56.

[18] Llinas-Regla J, Vilalta-Franch J, Lopez-Pousa S, Calvo-Perxas L, Torrents Rodas D, Garre-Olmo J. The Trail Making Test. Assessment Mar. 2017;24(2):183–96. 10.1177/1073191115602552. 10.1177/1073191115602552 26318386

[19] Goetzel RZ, Long SR, Ozminkowski RJ, Hawkins K, Wang S, Lynch W. Health, absence, disability, and presenteeism cost estimates of certain physical and mental health conditions affecting U.S. employers. J Occup Environ Med Apr. 2004;46(4):398–412. 10.1097/01.jom.0000121151.40413.bd. 10.1097/01.jom.0000121151.40413.bd 15076658

[20] Igarashi H, Ueda K, Jung S, Cai Z, Chen Y, Nakamura T. Social burden of people with the migraine diagnosis in Japan: evidence from a population-based cross-sectional survey. BMJ Open Nov. 2020;9(11):e038987. 10.1136/bmjopen-2020-038987. 10.1136/bmjopen-2020-038987 PMC7654137 33168555

[21] Stewart WF, Wood GC, Razzaghi H, Reed ML, Lipton RB. Work impact of migraine headaches. J Occup Environ Med Jul. 2008;50(7):736–45. 10.1097/JOM.0b013e31818180cb. 10.1097/JOM.0b013e31818180cb 18617829

[22] Stewart WF, Ricci JA, Chee E, Morganstein D, Lipton R. Lost productive time and cost due to common pain conditions in the US workforce. JAMA Nov. 2003;12(18):2443–54. 10.1001/jama.290.18.2443. 10.1001/jama.290.18.2443 14612481

[23] Ferrari MD. The economic burden of migraine to society. Pharmacoeconomics Jun. 1998;13(6):667–76. 10.2165/00019053-199813060-00003. 10.2165/00019053-199813060-00003 10179702

[24] Landy SH, Runken MC, Bell CF, Higbie RL, Haskins LS Assessing the impact of migraine onset on work productivity. J Occup Environ Med. 2011;53(1):74–81. . 10.1097/JOM.0b013e31812006365. 21187794 10.1097/JOM.0b013e31812006365

[25] Grodzka O, Dzagoevi K, Rees T, et al. Migraine with and without aura-two distinct entities? A narrative review. J Headache Pain Apr. 2025;14(1):77. 10.1186/s10194-025-01998-1. 10.1186/s10194-025-01998-1 PMC11995571 40229683

[26] Islamoska S, Hansen AM, Wang HX, et al. Mid- to late-life migraine diagnoses and risk of dementia: a national register-based follow-up study. J Headache Pain Aug. 2020;6(1):98. 10.1186/s10194-020-01166-7. 10.1186/s10194-020-01166-7 PMC7410151 32762715

[27] Gu L, Wang Y, Shu H. Association between migraine and cognitive impairment. J Headache Pain Jul. 2022;26(1):88. 10.1186/s10194-022-01462-4. 10.1186/s10194-022-01462-4 PMC9317452 35883043

