# Supplementary material for: Cognitive dysfunction during mild to moderate migraine attacks: potential implications for presenteeism
Source: BMC Neurol. 2026 Mar 4;26:234. doi: 10.1186/s12883-026-04782-z (PMC13067439; doi:10.1186/s12883-026-04782-z)
Supplement: Supplementary file 1 — Supplementary Material 1. [file 12883_2026_4782_MOESM1_ESM.docx]

Supplemental Table 1. Cognitive function assessment of HA (−) and HA (+) groups

|  | **HA (−)** | **HA (+)** | **Median difference**  **(HA (+) − HA (−)) (95% CI)** | **p value** |
| --- | --- | --- | --- | --- |
| N | 125 | 134 |  |  |
| D-CAT1 | 344 (300–397) | 326 (288–375) | −21 (−38 to −2) | 0.02 |
| D-CAT2 | 272 (235–298) | 252 (215–277) | −19 (−30 to −8) | 0.001 |
| D-CAT3 | 209 (181–242) | 191 (164–220) | -16 (−26 to −6) | 0.002 |
| TMT-A (s) | 25 (21–29) | 25 (22–31) | −1 (−3 to 1) | 0.26 |
| TMT-B (s) | 53 (45–64) | 51 (42–60) | 2 (−2 to 6) | 0.27 |

Mann–Whitney *U* test. Values are reported as median (IQR). Median differences and 95% confidence intervals were estimated using the Hodges–Lehmann method. D-CAT, Digit Cancellation Test; HA (−), without headache; HA (+), with headache; TMT, Trail Making Test.
