# Supplementary material for: Cognitive dysfunction during mild to moderate migraine attacks: potential implications for presenteeism
Source: BMC Neurol. 2026 Mar 4;26:234. doi: 10.1186/s12883-026-04782-z (PMC13067439; doi:10.1186/s12883-026-04782-z)
Supplement: Supplementary file 2 — Supplementary Material 2. [file 12883_2026_4782_MOESM2_ESM.docx]

Supplemental Table 2. Cognitive function assessment of HA (−) and HA (+) groups in patients aged ≤60 years

|  | **HA (−)** | **HA (+)** | **Median difference**  **(HA (+) − HA (−)), (95% CI)** | **p value** |
| --- | --- | --- | --- | --- |
| N | 120 | 134 |  |  |
| D-CAT1 | 350 (300–403) | 326 (288–375) | −22 (−40 to −4) | 0.02 |
| D-CAT2 | 274 (235–300) | 252 (215–277) | −21 (−31 to −9) | 0.001 |
| D-CAT3 | 209 (182–243) | 191 (164–220) | −18 (−28 to −7) | 0.001 |
| TMT-A (s) | 25 (21–29) | 25 (22–31) | −1 (−3 to 0) | 0.17 |
| TMT-B (s) | 52 (45–64) | 51 (42–60) | 1 (−2 to 5) | 0.44 |

Mann–Whitney *U* test. Values are reported as median (IQR). Median differences and 95% confidence intervals were estimated using the Hodges–Lehmann method. D-CAT, Digit Cancellation Test; HA (−), without headache; HA (+), with headache; TMT, Trail Making Test.
