# Supplementary material for: Cognitive dysfunction during mild to moderate migraine attacks: potential implications for presenteeism
Source: BMC Neurol. 2026 Mar 4;26:234. doi: 10.1186/s12883-026-04782-z (PMC13067439; doi:10.1186/s12883-026-04782-z)
Supplement: Supplementary file 3 — Supplementary Material 3. [file 12883_2026_4782_MOESM3_ESM.docx]

Supplemental Table 3. Cognitive function assessment of mild HA and moderate HA groups

|  | **Mild HA** | **Moderate HA** | **p value** |
| --- | --- | --- | --- |
| N | 79 | 55 |  |
| D-CAT1 | 327 (291–374) | 318 (279–376) | 0.58 |
| D-CAT2 | 250 (207–268) | 255 (227–281) | 0.29 |
| D-CAT3 | 193 (167–220) | 188 (162–223) | 0.81 |
| TMT-A (s) | 26 (23–30) | 24 (21–33) | 0.54 |
| TMT-B (s) | 53 (44–63) | 49 (41–56) | 0.21 |

Mann–Whitney *U* test. Values are reported as median (IQR). D-CAT, Digit Cancellation Test; HA, headache; TMT, Trail Making Test.
