# Supplementary material for: Cognitive dysfunction during mild to moderate migraine attacks: potential implications for presenteeism
Source: BMC Neurol. 2026 Mar 4;26:234. doi: 10.1186/s12883-026-04782-z (PMC13067439; doi:10.1186/s12883-026-04782-z)
Supplement: Supplementary file 4 — Supplementary Material 4. [file 12883_2026_4782_MOESM4_ESM.docx]

Supplemental Table 4. Cognitive function assessment of MO and MA in the HA (+) group

|  | **MO** | **MA** | **p value** |
| --- | --- | --- | --- |
| N | 112 | 22 |  |
| D-CAT1 | 319 (281–374) | 340 (309–385) | 0.16 |
| D-CAT2 | 255 (214–280) | 246 (218–268) | 0.62 |
| D-CAT3 | 191 (165–220) | 189 (162–220) | 0.79 |
| TMT-A (s) | 25 (22–31) | 25 (20–30) | 0.56 |
| TMT-B (s) | 51 (42–61) | 48 (42–58) | 0.89 |

Mann–Whitney *U* test. Values are reported as median (IQR). D-CAT, Digit Cancellation Test; HA, headache; MA, migraine with aura; MO, migraine without aura, TMT, Trail Making Test.
